# Exploratory high *b* value diffusion-weighted MR for quantitative differentiation of ileocecal inflammatory conditions and tumors

**DOI:** 10.1186/s13244-025-01916-8

**Published:** 2025-02-17

**Authors:** Hao Yu, Yucheng Hai, Jingyu Lu

**Affiliations:** 1 Department of Radiology, Zhongnan Hospital of Wuhan University, Wuhan, China; 2 Department of Radiology, Tongji Hospital, Tongji Medical College, Huazhong University of Science and Technology, Wuhan, China

**Keywords:** Diffusion-weighted imaging, High *b* value, Tumor, Inflammation

## Abstract

**Objectives:**

To explore the quantitative analysis of high *b* value (2000 s/mm^2^) diffusion-weighted imaging (DWI) for the differentiation of ileocecal inflammatory conditions and tumors, compared with conventional *b* value (800 s/mm^2^) DWI.

**Methods:**

Sixty-six patients with 30 tumors and 36 inflammatory conditions underwent MR enterography with conventional and high *b* values DWI. Quantitative apparent diffusion coefficient (ADC) values and signal intensity ratios (SIRs) of lesions of the psoas muscle were measured from the two *b* value DWIs. The receiver operating characteristic (ROC) curve was applied to determine the diagnostic value of ADC and SIR for differentiating tumors from inflammatory conditions.

**Results:**

The ADC values of tumors were significantly lower than those of inflammatory conditions in 800 s/mm^2^ (*p* = 0.001) and 2000 s/mm^2^ (*p* < 0.001) DWI. In addition, tumors exhibited significantly higher SIR values compared to inflammatory conditions (*p* < 0.001 in 800 s/mm^2^ and 2000 s/mm^2^ DWI). Areas under the curve (AUC) of ADC and SIR derived from high *b* value (0.828 for ADC, 0.947 for SIR) were superior to those from conventional *b* value DWI (0.731 and 0.849, respectively). Compared to ADC, SIR values achieved better AUCs in both two *b* values DWI.

**Conclusions:**

Quantitative ADC values and SIR could be used as non-invasive tools to distinguish ileocecal tumors from inflammatory conditions. The use of high *b* value DWI would improve this ability. Furthermore, SIR obtained from high *b* value DWI was the most promising quantitative parameter.

**Critical relevance statement:**

This study indicated that quantitative DWI parameters might be applied as non-invasive imaging biomarkers for distinguishing bowel tumors from inflammatory conditions. The SIR from high *b* value DWI could improve the differentiation, providing invaluable information for establishing appropriate therapeutic strategies.

**Key Points:**

- Differentiation between bowel inflammatory conditions and tumors is still a dilemma.
- Quantitative DWI contributed to distinguishing ileocecal tumors from inflammatory conditions.
- SIR from DWI is a promising parameter for differentiating these pathologies.

**Graphical Abstract:**

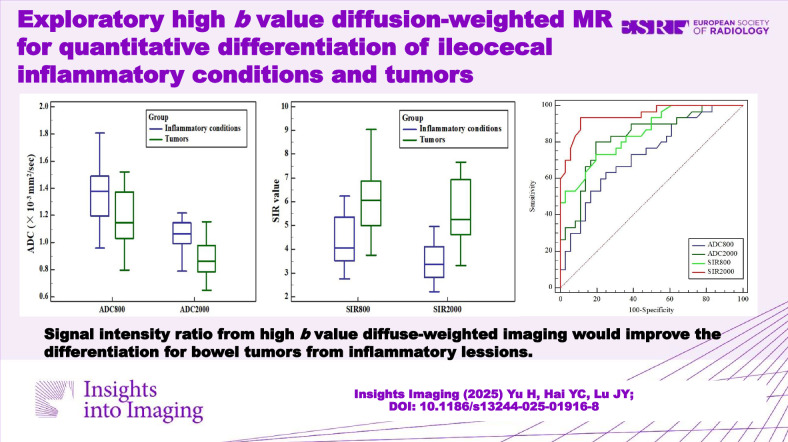

## Introduction

Recently, MR enterography (MRE) has been increasingly utilized not only for the assessment of bowel inflammations but also for the evaluation of bowel tumors [1–4]. However, differentiation between bowel inflammatory conditions and tumors is challenging due to their considerable overlap in clinical symptoms and imaging features [3, 5]. In addition, patients with chronic and prolonged bowel inflammation are at a greater risk of intestinal tumors compared to the general population [3, 5, 6]. It is crucial to accurately differentiate bowel tumors from inflammatory conditions for the appropriate and prompt clinical intervention [3–6].

As a promising and functional technique reflecting the random movement of water molecules in biological tissues, diffusion-weighted imaging (DWI) is widely applied to evaluate inflammatory conditions and tumors of abdominal organs [7–9]. In addition, DWI is easily inserted into clinical MR examination schemes, providing more information for the detection and identification of lesions in clinical settings [8–10]. Meanwhile, quantitative parameters of apparent diffusion coefficient (ADC) and signal intensity ratio (SIR) from DWI have emerged as the objective modalities for differentiating various pathological conditions [11–14]. However, both bowel inflammatory conditions and tumors can exhibit hyperintensity on the conventional *b* value DWI, which makes the differentiation between these two pathologies challenging [3, 5, 7, 15]. Recently, high *b* value DWI has been found useful for the identification of different pathological conditions or tumor grades [14, 16–18]. A previous study has shown that high *b* value DWI can suppress the signal intensity of tumor-associated acute inflammation and help delineate pancreatic adenocarcinomas [19].

Thus, this study aimed to compare the performances of quantitative parameters derived from conventional and high *b* value DWI to differentiate ileocolonic tumors from inflammatory conditions, considering endoscopic or postoperative histopathological examinations as the golden standard.

## Method

### Patient selection

**Fig. 1:**
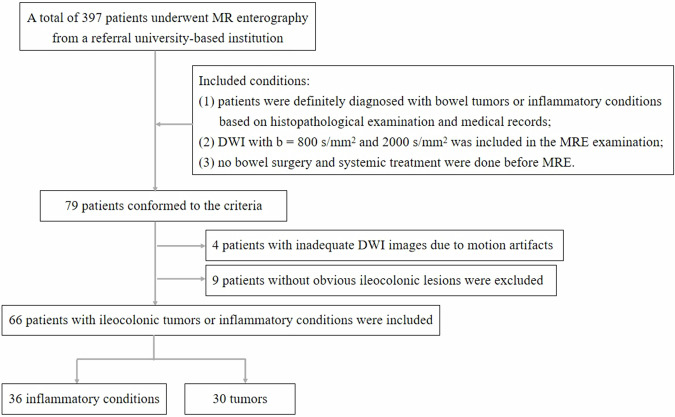
Flow chart of the study

The institutional review board approved this retrospective study and waived informed consent. By searching our database, we identified 397 patients who underwent MRE in the referral university-based institution. Seventy-nine patients conformed to the following criteria: (1) patients were definitely diagnosed with bowel tumors or inflammatory conditions based on histopathological examination and medical records; (2) DWI with conventional *b* value (*b* = 800 s/mm^2^) and high *b* value (*b* = 2000 s/mm^2^) were conducted in the MRE examination; (3) no bowel surgery and systemic treatment were done before MRE. We excluded four patients with inadequate DWI images due to motion artifacts and nine patients without obvious ileocolonic lesions. Finally, 66 patients with ileocolonic tumors or inflammatory conditions were included in this study (Fig. 1).

### MRE data acquisition

MRE examinations were conducted using a 3.0-T MR scanner (Discovery MR750, GE Healthcare) in the supine position with a 32-channel torso-phased array body coil. All patients fasted for at least 6 h before MRE. Approximately 1500 mL of 2.5% mannitol solution was administered orally to the patients 60 min before MR scanning to achieve adequate distension of the bowel. Anisodamine 20 mg was administered intramuscularly 5 min before MRE to decrease bowel peristalsis. Conventional MRE sequences were acquired, including T2-weighted single-shot fast spin-echo (TR/TE: a respiratory cycle/68 ms, matrix: 288 × 288, slice thickness: 4 mm for coronal/5 mm for axial), the fast imaging employing steady-state acquisition (TR/TE: 3.2/1.2 ms, matrix: 288 × 288, slice thickness: 4 mm for coronal/5 mm for axial, flip angle: 45°), and coronal and axial T1 weighted three-dimensional fast spoiled gradient echo (liver acquisition with volume acceleration sequence; TR/TE: 3.8/1.7 ms, matrix: 260 × 210, slice thickness: 4 mm, flip angle: 15°).

DWI sequences were acquired in the transverse plane by free-breathing single-shot echo planar imaging (SS-EPI, TR/TE: 4200 ms/minimum, field of view: 38.0 × 30.4 cm, matrix: 160 × 128, slice thickness: 6 mm, flip angle, 90°) with *b* values of 800 s/mm^2^ and 2000 s/mm^2^. The number of excitations was 6 for the 800 s/mm^2^ *b* value and 10 for the 2000 s/mm^2^ *b* value. Frequency-selective fat saturation was used to prevent chemical shift artifacts, and the sensitivity encoding acceleration factor was 2.

### MR image analysis

All images were transferred to a workstation (AW4.6, GE Healthcare). ADC maps were generated from DWI at 2 different *b* values (0 s/mm^2^ and 800 s/mm^2^ for the ADC_800_ map; and 0 s/mm^2^ and 2000 s/mm^2^ for the ADC_2000_ map) using a mono-exponential fit. Two gastrointestinal radiologists conducted the quantitative analysis of DWI (R1 and R2 with 5 years and 8 years of experience, respectively). They were blinded to clinical data and pathological findings. Three oval regions of interest (ROI) were drawn on the brightest area around the lesion on DWI with *b* values of 800 s/mm^2^ or 2000 s/mm^2^ and copied to the corresponding ADC maps. During the measurement process, any cystic changes, necrotic areas, abscess cavities, and fistulas of lesions identified in corresponding DWI and conventional T1/T2 images were excluded. Simultaneously, the signal intensity of the lesions (SI_lesion_) and ADC values were recorded. The mean value of three measurements (SI_lesion_ and ADC) was used for further analysis. The signal intensity of the psoas muscle (SI_muscle_) was also measured by placing a larger oval ROI on corresponding images from 800 s/mm^2^ or 2000 s/mm^2^ DWI. Based on previous studies, the SIR of the lesion to muscle was calculated using the equation: SIR = SI_lesion_/SI_muscle_ [13, 20, 21].

### Statistical analysis

Statistical analyses were conducted using the Statistical Package for the Social Sciences Software Package (SPSS Statistics, version 26.0; SPSS Inc., Chicago, IL) and MedCalc (Version 20.006, MedCalc Software, Mariakerke, Belgium). Inter-observer agreement for measuring SIR and ADC was determined using the intraclass correlation coefficient (ICC). The means of ADC and SIR between two radiologists were used for further analyses. Differences in categorical variables between the tumor group and the inflammatory condition group were compared using the chi-square test. The normality of quantitative ADC and SIR was ascertained using the Kolmogorov-Smirnov test. ADC and SIR between the inflammatory conditions and tumors or between two *b* values DWI were compared using the independent *t*-test. Diagnostic efficiencies of DWI parameters were determined by the receiver operating characteristic (ROC) curve. The area under ROC (AUC) was compared between SIR and ADC using the method defined by Delong et al Statistical significance was defined as *p* < 0.05.

## Results

### Study population

**Fig. 2:**
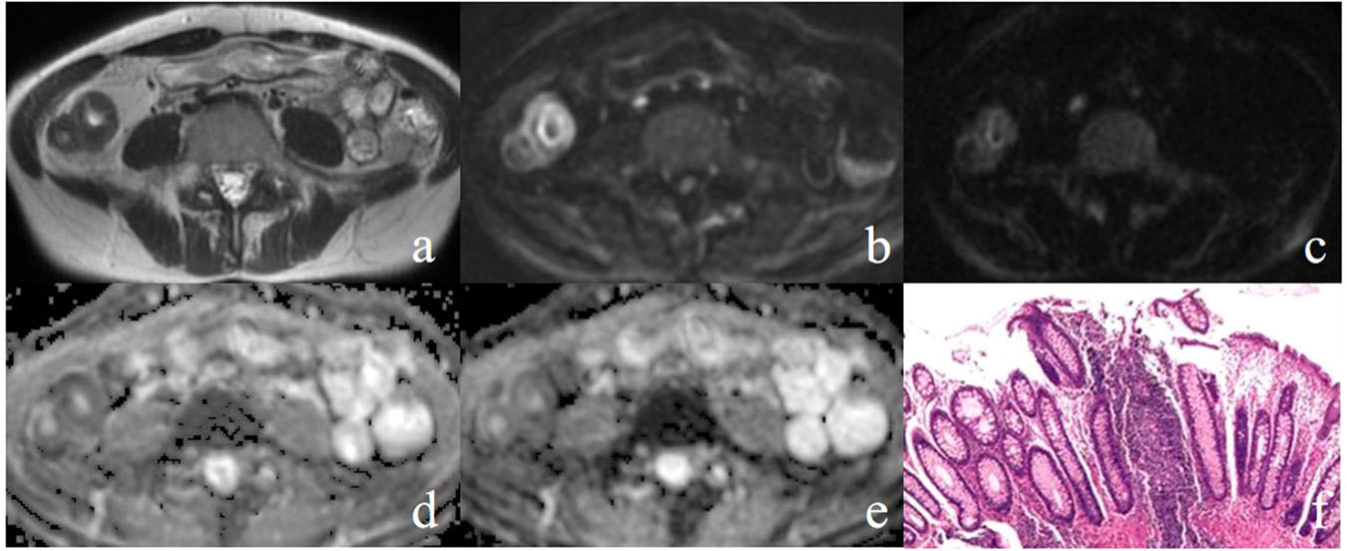
Terminal ileum of a 41-year-old female with Crohn’s disease demonstrated wall thickening on axial T2WI MRE (**a**). It presented hyperintensity on DWI with a *b* value of 800 s/mm^2^ (**b**). The signal intensity of the ileal lesion was significantly decreased on DWI at 2000 s/mm^2^ DWI (**c**). Terminal ileum showed hypointensity on ADC map from DWI with *b* = 800 (**d**) and 2000 s/mm^2^ (**e**). Histopathology confirmed neutrophil infiltration (**f**)

**Fig. 3:**
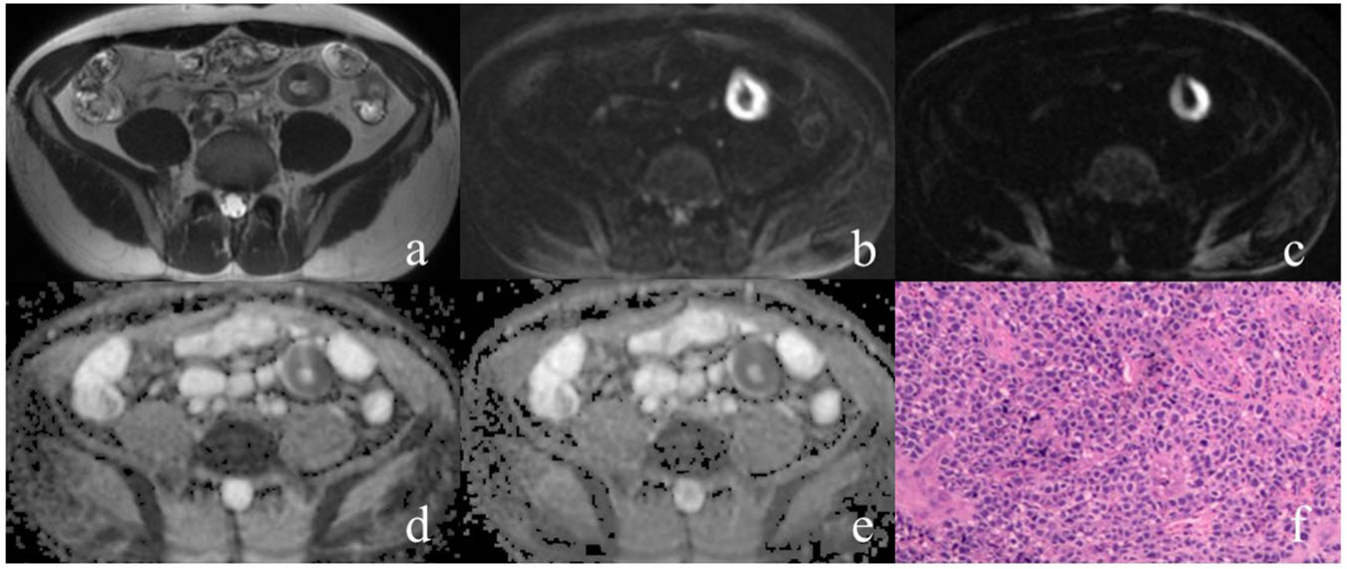
A 41-year-old man with distal ileal lymphoma. The distal ileum wall was thickened on axial T2WI (**a**). The lesion exhibited relatively high signal intensity on DWI with *b* = 800 (**b**) and 2000 s/mm^2^ (**c**), and presented hypointensity on corresponding ADC_800_ (**d**) and ADC_2000_ map (**e**). Histopathology revealed the amount of lymphocytic tumor cells in the tissue (**f**)

Finally, 66 patients (42 men and 24 women) were included in this study, with a mean age of 42.7 ± 15.4 years. Based on endoscopic or postoperative histopathological findings and clinical records, 36 patients were diagnosed with inflammatory conditions (19 patients with Crohn’s disease, 11 patients with intestinal tuberculosis, and 6 patients with ulcerative colitis). The remaining 30 patients were confirmed to have intestinal tumors, including 22 patients with adenocarcinomas and 8 patients with lymphomas. Older age was found in patients with tumors compared to those with inflammatory conditions (*p* < 0.001). There were no significant differences between the two groups in terms of sex, lesion morphology, enlarged adjacent lymph node, and proximal bowel expansion (Table 1). The typical inflammatory and tumorous lesions are shown in Figs. 2 and 3. As compared to adenocarcinomas, lymphomas exhibited slightly greater SIR and lower ADC without significant difference in both 800 s/mm^2^ and 2000 s/mm^2^ DWI (SIR_800_: 6.66 ± 1.41 vs 5.94 ± 1.40 *p* = 0.225, ADC_800_: 1.08 ± 0.18 vs 1.22 ± 0.19 *p* = 0.073, SIR_2000_: 6.19 ± 1.21 vs 5.40 ± 1.22 *p* = 0.129, ADC_2000_: 0.83 ± 0.12 vs 0.90 ± 0.13 *p* = 0.172, ADC unit: × 10^−3^ mm^2^/s), the mean values of ADC and SIR from 22 adenocarcinomas and 8 lymphomas were compared with inflammatory conditions.

**Table 1:** Clinical data of patients’ population

Clinic characteristic	Inflammatory conditions (*n* = 36)	Tumors (*n* = 30)	*p* value
Age (years)	35.1 ± 10.5 [range: 21–59]	51.9 ± 17.6 [range: 28–77]	< 0.001
Sex (male:female)	24:12	18:12	0.761
Lesions morphology			
Wall thickness	22 (61.1%)^‡^	14 (46.7%)^‡^	0.355
Local mass	14 (38.9%)^‡^	16 (53.3%)^‡^	
Enlarged adjacent lymph nodes (> 1 cm)	13 (36.1%)^‡^	17 (56.7%)^‡^	0.155
Proximal bowel expansion (> 3 cm)	10 (27.8%)^‡^	14 (46.7%)^‡^	0.183

^‡^ The data were expressed as numbers (percentages)

### Diagnostic efficiencies of ADC and SIR

**Fig. 4:**
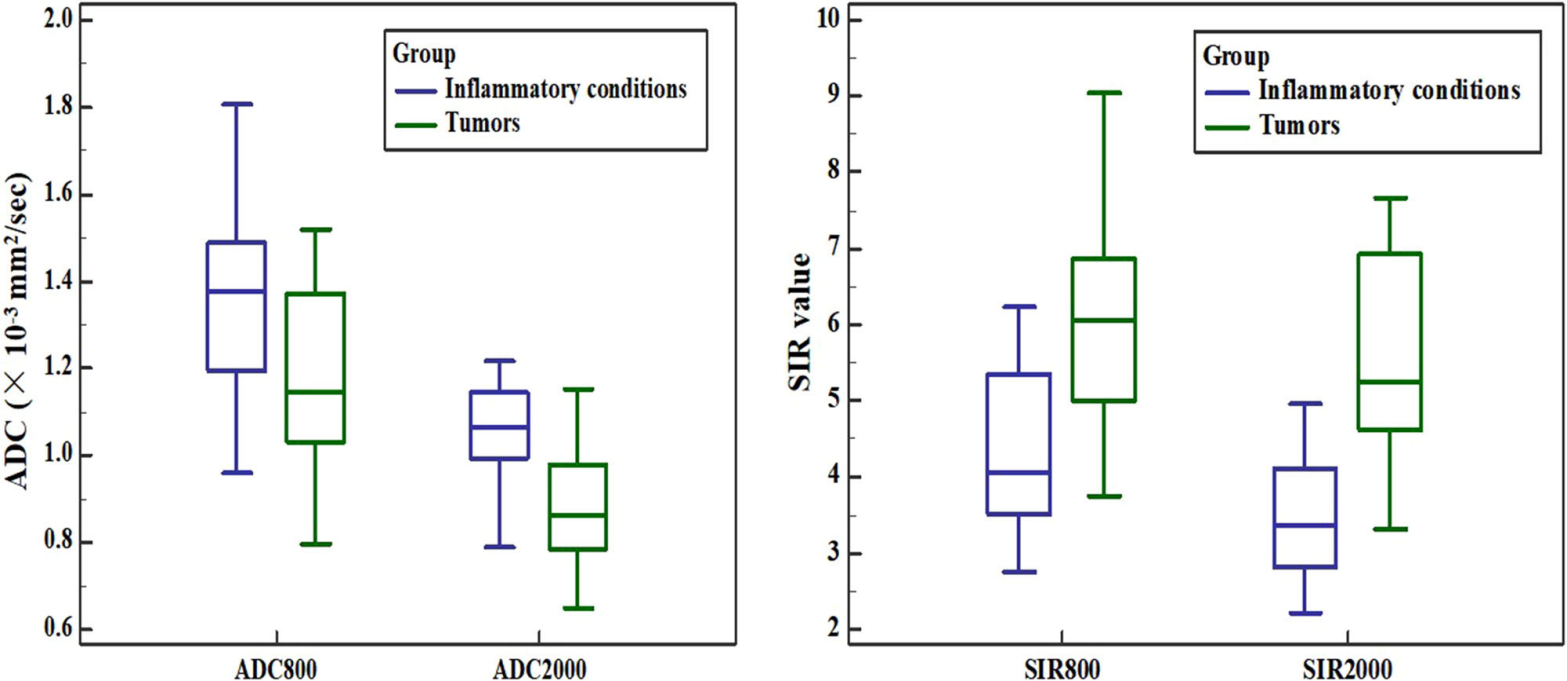
Box-and-whisker of ADC and SIR values from 800 s/mm^2^ and 2000 s/mm^2^ DWI between inflammatory conditions and tumors

The mean values of ADC_800_ and ADC_2000_ were significantly lower in tumors than in inflammatory conditions (*p* = 0.001 for ADC_800_ and *p* < 0.001 for ADC_2000_), as shown in Table 2 and Fig. 4. With the cut-off value of 1.20 × 10^−3^ mm^2^/s for ADC_800_ and 0.98 × 10^−^^3^ mm^2^/s for ADC_2000_, the sensitivities of them were 63.3%, 75.0%, and the specificities were 75.0%, 80.6%, respectively. The sensitivity of ADC value was greater for higher *b* value compared to conventional *b* value DWI.

**Table 2:** Diagnostic efficiencies of ADC and SIR from two *b* values DWI for lesions differentiation

Parameters	Inflammatory conditions	Tumors	Cut-off value	*p* value	AUC	Sensitivity (%)	Specificity (%)
ADC_800_	1.36 ± 0.20 [1.29–1.43]	1.18 ± 0.19 [1.11–1.26]	1.20	0.001	0.731	63.3	75.0
ADC_2000_	1.05 ± 0.12 [1.01–1.09]	0.88 ± 0.13 [0.83–0.93]	0.98	< 0.001	0.828	80.0	80.6
SIR_800_	4.31 ± 1.05 [3.96–4.67]	6.13 ± 1.42 [5.60–6.66]	5.54	< 0.001	0.849	73.3	80.6
SIR_2000_	3.45 ± 0.79 [3.18–3.72]	5.61 ± 1.25 [5.15–6.08]	4.39	< 0.001	0.947	93.3	88.9

The values in square brackets are 95% confidence intervals

ADC unit: ×10^−^^3^ mm^2^/s

*ADC* apparent diffusion coefficient, *SIR* signal intensity ratio, *DWI* diffusion-weighted imaging, *AUC* areas under receiver operating characteristic

The SIR values demonstrated superb sensitivities and specificities in DWI with a *b* value of 800 s/mm^2^ (73.3% and 80.6%) and 2000 s/mm^2^ (93.3% and 88.9%). In particular, they exhibited significantly higher values in tumors than in inflammatory conditions (*p* < 0.001 for both 800 and 2000 s/mm^2^ DWI). Similarly, SIR values achieved better sensitivity and specificity in 2000 s/mm^2^ compared with those in 800 s/mm^2^ DWI (Table 2 and Fig. 4).

### Comparisons of AUC for ADC and SIR values from two *b* values DWI

**Fig. 5:**
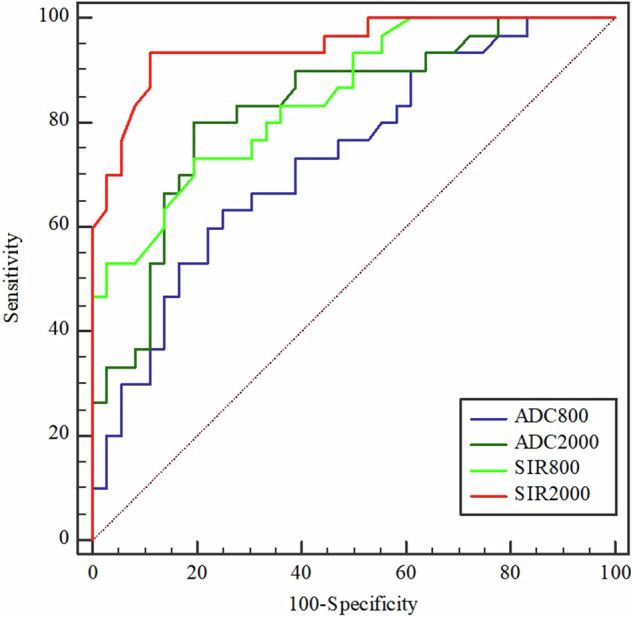
AUCs of ADC and SIR values from 800 s/mm^2^ to 2000 s/mm^2^ DWI

The AUCs of ADC_800_, ADC_2000_, SIR_800_, and SIR_2000_ for the differentiation of tumors and inflammatory conditions were 0.731, 0.828, 0.849, and 0.947, respectively. The results of AUC comparisons are presented in Table 3 and Fig. 5. The AUCs of ADC and SIR from DWI with a *b* value of 2000 s/mm^2^ were significantly increased compared to those from DWI with *b* = 800 s/mm^2^ DWI (*p* value: 0.024 for ADC, 0.020 for SIR). Compared to ADC values, SIR values achieved higher AUCs in both *b* values DWI (*p* value: 0.042 for 800 s/mm^2^, 0.026 for 2000 s/mm^2^).

**Table 3:** AUC comparisons of ADC and SIR values from the two *b* values DWI

AUC comparisons	*p* value
ADC_800_ VS ADC_2000_	0.024
SIR_800_ VS SIR_2000_	0.020
ADC_800_ VS SIR_800_	0.042
ADC_2000_ VS SIR_2000_	0.026

*AUC* areas under receiver operating characteristic, *ADC* apparent diffusion coefficient, *SIR* signal intensity ratio, *DWI* diffusion-weighted imaging

### Comparisons of ADC and SIR values between 800 s/mm^2^ and 2000 s/mm^2^ DWI within the inflammatory condition group or tumor group

Table 4 presents the comparisons of ADC and SIR values between the two *b* values DWI. The ADCs of inflammatory conditions and tumors both decreased when *b* = 2000 s/mm^2^ compared to those when *b* = 800 s/mm^2^ DWI (*p* < 0.001). SIR_2000_ values of inflammatory conditions were significantly lower than SIR_800_ values of inflammatory conditions (*p* < 0.001). However, there was no significant difference between SIR_2000_ and SIR_800_ of tumors (*p* = 0.138).

**Table 4:** Comparisons of ADC and SIR values between 800 s/mm^2^ and 2000 s/mm^2^ DWI

Group	ADC_800_	ADC_2000_	*p* value	SIR_800_	SIR_2000_	*p* value
Inflammatory conditions	1.36 ± 0.20	1.05 ± 0.12	< 0.001	4.31 ± 1.05	3.45 ± 0.79	< 0.001
Tumors	1.18 ± 0.19	0.88 ± 0.13	< 0.001	6.13 ± 1.42	5.61 ± 1.25	0.138

ADC unit: × 10^−^^3^ mm^2^/s

*ADC* apparent diffusion coefficient, *SIR* signal intensity ratio, *DWI* diffusion-weighted imaging

### Interobserver agreement analysis

ICCs between the two radiologists were 0.807 (95% CI: 0.704–0.878) for ADC and 0.885 (95% CI: 0.819–0.928) for SIR in 800 s/mm^2^ DWI. ICCs for ADC_2000_ and SIR_2000_ were 0.868 (95% CI: 0.793–0.917) and 0.908 (95% CI: 0.855–0.943), respectively.

## Discussion

In this study, the quantitative ADC and SIR derived from DWI were found effective for the differentiation of bowel inflammatory conditions and tumors. The diagnostic yields of ADC and SIR were significantly improved, when a high *b* value (2000 s/mm^2^) was applied. Besides, quantitative SIR was more valuable than ADC in both *b* values DWI. Therefore, SIR calculated from high *b* value images may be a valuable and promising parameter for distinguishing tumors from inflammatory conditions.

Due to the development of fast image acquisition techniques, gradient coils, and improvements in MR hardware, high *b* value DWI is increasingly used in clinical practice, showing great values for lesion differentiation and tumor classification [16–18, 22]. Previous studies have shown that ADC values obtained from DWI with a high *b* value of 1500–3000 s/mm^2^ had better diagnostic performance compared to conventional *b* value DWI for the differential diagnosis of brain lesions [16, 17], breast cancers [23], and prostate tumors [18, 24]. Our study demonstrated similar results, with significantly better AUCs for *b* = 2000 s/mm^2^ compared to those for *b* = 800 s/mm^2^ DWI. Just as in the study of ADC analysis in liver lesions [25], ADC values of inflammatory conditions and tumors were both decreased in the high *b* value DWI group. This phenomenon was associated with the decreased microperfusion in tissues and the T2 shine-through effect on the high *b* value DWI [14, 17, 19]. In addition, compared to directly acquired DWI, computed or synthetic high *b* value DWI (cDWI), generated from two or more lower *b* values images through mathematical calculation, improved the conspicuity of small ischemic cerebral lesions and achieved excellent visualization of the prostate and pancreas cancers [26–28]. Furthermore, cDWI with high *b* values is useful for differentiating benign and malignant gallbladder lesions without additional scanning time [29]. Further exploration of cDWI in intestines is of great value for clinical application.

The signal intensity and image contrast of DWI mainly rely on the selected *b* value [14]. High *b* value DWI effectively suppressed the signal intensity of backgrounds and benign lesions, demonstrating great advantages for detecting tumors and malignancies through visual assessment [19, 30, 31]. Similarly, the signal intensity of inflammatory conditions was significantly decreased on high *b* value DWI, while intestinal tumors still demonstrated relatively hyperintensity. The quantitative SIR values of the inflammatory conditions were dramatically reduced when *b* values increased from 800 s/mm^2^ to 2000 s/mm^2^. While the differences in SIR from tumors between 800 s/mm^2^ and 2000 s/mm^2^ DWI were not significantly different. This phenomenon might be attributed to the higher cell density in tumor lesions. Therefore, DWI with a high *b* value demonstrated a better image contrast between inflammatory conditions and tumors. SIR from DWI with a *b* value of 2000 s/mm^2^ exhibited the optimal diagnostic performance in our study.

In addition, SIR was found to be superior to ADC for distinguishing tumors from inflammatory conditions in both 800 s/mm^2^ and 2000 s/mm^2^ DWI from the current study. The diagnostic yields of SIR were better, probably because calculating SIR based on the same *b* value DWI image limited motion degradation, as the acquisition of a single *b* value DWI image using SS-EPI is rapid and sub-second using the latest accelerated acquisition techniques and multi-channel coils [32, 33]. As is known, ADC values derived from DWI images with at least two *b* values can be affected by image mismatch, eddy currents, distortion, and motion in images of different *b* values, especially for hollow organs, such as the gastrointestinal tract or gallbladder [8, 34]. Therefore, the SIR can more precisely represent the tissue diffusion condition on DWI, and these results are worth further study.

There are several limitations to this study that should be acknowledged. Firstly, it was a retrospective study with a relatively small and unbalanced sample size in the inflammatory conditions and tumor groups. Further prospective trials with larger sample sizes can help confirm these results. Then, as the thin normal bowel wall may be invisible on DWI images, the psoas muscle was used as the reference for calculating the SIR. Additionally, 2D ROI placement on the bowel wall was relatively challenging for observers. Improvements in automated approaches to ROI placement and 3D VOI containing all the lesions are of great value.

## Conclusions

The quantitative ADC and SIR from DW-MRE were useful for non-invasive differentiation between ileocecal tumors and inflammatory conditions. High *b* value DWI improved the diagnostic yields of ADC and SIR. Furthermore, SIR was superior to ADC values for differentiation between ileocecal tumors and inflammatory conditions. Therefore, SIR derived from a high *b* value DWI could be a valuable and promising parameter.

## Data Availability

The data underlying this article are available from the corresponding author on reasonable request.

## Abbreviations

ADC: Apparent diffusion coefficient
AUC: Areas under the curve
DWI: Diffusion-weighted imaging
ICC: Intraclass correlation coefficient
MRE: MR enterography
SI_lesion_: Signal intensity of the lesions
SI_muscle_: Signal intensity of psoas muscle
SIR: Signal intensity ratio
SS-EPI: Single shot echo planar imaging
